# Targeting Metastatic Colorectal Cancer with Immune Oncological Therapies

**DOI:** 10.3390/cancers13143566

**Published:** 2021-07-16

**Authors:** Norman J. Galbraith, Colin Wood, Colin W. Steele

**Affiliations:** 1 Academic Department of Surgery, University of Glasgow, Level 2 New Lister Building, Glasgow Royal Infirmary, 10-16 Alexandra Parade, Glasgow G31 2ER, UK; colin.wood@glasgow.ac.uk (C.W.); colin.steele@glasgow.ac.uk (C.W.S.); 2 Institute of Cancer Sciences, Beatson Institute, Garscube Campus, Switchback Road, Bearsden G61 1BD, UK

**Keywords:** colorectal cancer, immunotherapy, metastases, immune checkpoint inhibitors, anti-PD1, tumour microenvironment, targeted therapy, microsatellite instability, mismatch repair, tumour-associated macrophages

## Abstract

**Simple Summary:**

Colorectal cancer that has become metastatic continues to have a poor outlook. Many patients will undergo intensive chemotherapy, often with limited effects but many side effects. In other types of cancer, immunotherapy has shown to be effective by targeting the immune cells of the patient and restoring their function to fight off cancer. This review explores the up-to-date evidence for immunotherapy in metastatic colorectal cancer. We have discussed in which patients this treatment is effective, but also why this has not been effective in a large number of patients. By summarizing the key components of the immune cells within the proximity to the tumour and its areas of spread, we discuss how these components can be targeted. Areas of future research are also highlighted, which includes combining immunotherapy with current treatments, such as surgery, radiotherapy and chemotherapy as well as some recent advances from basic and translational studies that promises to improve outcomes in these patients.

**Abstract:**

Metastatic colorectal cancer carries poor prognosis, and current therapeutic regimes convey limited improvements in survival and high rates of detrimental side effects in patients that may not stand to benefit. Immunotherapy has revolutionised cancer treatment by restoring antitumoural mechanisms. However, the efficacy in metastatic colorectal cancer, is limited. A literature search was performed using Pubmed (Medline), Web of Knowledge, and Embase. Search terms included combinations of immunotherapy and metastatic colorectal cancer, primarily focusing on clinical trials in humans. Analysis of these studies included status of MMR/MSS, presence of combination strategies, and disease control rate and median overall survival. Evidence shows that immune checkpoint inhibitors, such as anti-PD1 and anti-PD-L1, show efficacy in less than 10% of patients with microsatellite stable, MMR proficient colorectal cancer. In the small subset of patients with microsatellite unstable, MMR deficient cancers, response rates were 40–50%. Combination strategies with immunotherapy are under investigation but have not yet restored antitumoural mechanisms to permit durable disease regression. Immunotherapy provides the potential to offer additional strategies to established chemotherapeutic regimes in metastatic colorectal cancer. Further research needs to establish which adjuncts to immune checkpoint inhibition can unpick resistance, and better predict which patients are likely to respond to individualised therapies to not just improve response rates but to temper unwarranted side effects.

## 1. Introduction

Colorectal cancer (CRC) is the second greatest cancer threat to life in the Western World [1]. Very few advances have been made in targeting metastatic colorectal cancer (mCRC) effectively. CALGB/SWOG 80405 [2], Fire-3 [3] and PEAK [4] randomised controlled trials have all firmly established bevacizumab (VEGFR-inhibitor), cetuximab and panitumumab, (EGFR inhibitors) as first-line therapies in addition to FOLFOX/FOLFOXIRI in the management of patients with metastatic colorectal cancer. However, the margins of benefit in addition to standard therapies are minimal, with progression-free survival in FIRE-3 enhanced from 5.8 months to 9.7 months with the addition of cetuximab in the truly RAS wild-type patients [4]. These responses are in contrast to those seen in other malignancies, such as melanoma, where in BRAF mutant cancer a combination of BRAF and MEK inhibition with immunotherapy has revolutionised treatment and led to survival for patients extending to a median of 2 years, from less than 9 months [5].

However, hope exists for patients with colorectal cancer that targeted therapies can start to be applied to specific subgroups of patients. The recently coordinated BEACON study on the failure of BRAF directed inhibition alone in *BRAFV600E* mutant patients sought to address the role of EGRF pathway reactivation in resistance to therapy. Using a combination of standard chemotherapy, encorafenib (BRAF inhibitor) and MEK inhibitor, binimetinib, significantly improved survival in this dismal prognosis disease [6]. It is important to recognise this only applies to 10% of patients with colorectal cancer, and in late-stage disease, outcomes remain <10 months even with targeted therapy.

Despite these recent advances, surgery provides the potential for cure in patients with metastatic disease. However, sadly most patients are not candidates for resection due to the burden or distribution of disease. Colorectal cancer oncological therapies must look to capitalise on the successes achieved in other cancers with immunotherapies to enhance patient survival, combining these in the future where possible with surgical resection to benefit patients with lower volume oligometastatic disease. An opportunity exists to bring forward treatments that are being used in the most advanced disease to provide opportunities to achieve long-term survival in more patients with borderline resectable/resectable metastatic disease. This review will focus on the mutational and transcriptomic landscape of colorectal cancer and current understanding of drug positioning in disease subtypes. We will illustrate previous and current trials of immune oncological agents in mCRC and explore avenues for combinatorial immune oncological approaches in this disease.

## 2. Colorectal Cancer—Personalised Medicine for a Heterogeneous Disease

Patients presenting with colorectal cancer can be classified by stage of disease. Patients are increasingly presenting early through screening with pre-cancerous polyps or localised disease (stages 0–III). However, there are still a significant proportion of patients that present with stage IV disease, which is frequently metastatic and not surgically operable. Whilst local excision, major colorectal resection and metastasectomy have an important role to play, it is the patients with metastatic colorectal cancer (mCRC) in whom systemic therapeutic strategies offer poor response rates and limited potential for disease control. Increasing understanding of major genotypic and phenotypic variation in colorectal cancer permits a tailored strategy to help predict likely responses to a given agent, which maximises the benefit whilst minimising the suffering from side effects and toxicity in patients who do not stand to benefit.

The most common mutations that are observed in colorectal tumours include APC (40%), TP53 (65%), KRAS (50%) and SMAD4 (12%), amongst many others [7,8]. The majority of colorectal cancers occur via the adenoma–carcinoma sequence, with an accumulation of mutations occurring over time [9]. Until recently, chemotherapy for metastatic colorectal cancer has included a combination of oxaliplatin, irinotecan, fluorouracil (5-FU) and capecitabine, such as FOXFOX, FOLFIRI, FOLFIRINOX and CAPOX [10]. It is on the basis of certain mutations in which targeted therapy has begun to be explored in the treatment of colorectal cancer.

### 2.1. Classification by Mutational Status of CRC

B-type RAF (BRAF) mutations occur in 5–15% of mCRC and are associated with right-sided colon cancer [11]. Patients who have BRAF-mutant mCRC (V600E being the most common mutation) are generally treated with FOLFOXIRI plus bevacizumab (monoclonal antibody against VEGF-A) [10]. There is some controversy over the effectiveness of anti-EGR therapies in patients with wild-type RAS but BRAF mutant tumours. A meta-analysis by Rowland et al. concluded that the BRAF mutation status did not significantly affect treatment benefit and that there was insufficient data to exclude patients with BRAF mutations from anti-EGFR therapy [12]. This was in contrast to Pietrantonio et al., who concluded that anti-EGFR therapy did not have any benefit beyond standard therapy in patients with RAS wild-type, BRAF mutant colorectal cancer [13]. More recently, the VOLFI study confirmed that the addition of panitumumab to mFOLFOXIRI in patients with RAS wild-type metastatic CRC improved the ORR and indeed the rate of secondary resection for metastases [14]. RAS mutations occur in approximately half of patients with colorectal cancer and also affect the RAS-RAF-ERK signalling pathway [15]. Patients with RAS activating mutations also have poorer prognosis and, such as BRAF, are predicted to have resistance to anti-EGFR therapies, such as cetuximab and panitumumab [16]. Assessing the mutation status of patients for BRAF and RAS mutant tumours allows targeted strategies to maximise effectiveness and minimise toxicity. In patients who do not respond to anti-EGFR therapy, MEK inhibitors are under investigation to target downstream effector pathways (RAS-RAF-MEK-MAPK) to limit proliferation and promote tumoural apoptosis to inhibit tumour growth [17]. It is of note that one trial is underway studying the role of cetuximab and pembrolizumab in RAS-wild-type patients with colorectal cancer [18].

Mismatch repair (MMR) deficiency occurs through mutations or promoter hypermethylation in MLH1, MSH2, MSH6 or PMS2 [19]. Due to a failure of these genes to repair base–base mismatches during DNA replication, decreased genomic stability and progression to colorectal cancer occurs. As a result of this defect, large mutations at microsatellite sequences are observed, termed microsatellite instability (MSI). Colorectal cancer specimens undergo immunohistochemistry (IHC) as standard to assess MMR status, and PCR to determine MSI status [20]. Fifteen percent of colorectal cancers are considered mismatch repair deficient, microsatellite instability-high (dMMR-MSI-H) tumours [19,20]. Whilst traditional chemotherapy is less effective, these patients have a better prognosis. As a result of the better prognosis, only around 5% of those with metastatic CRC are dMMR-MSI-H tumours. In patients that have a mismatch-repair deficient tumour, there is a high tumour mutational burden (TMB) (more than 12 mutations per 10^6^ DNA bases) which offers a high number of neoantigens which are recognised as foreign by the local patrolling immune cells [19,21]. As a result, largely due to an upregulated T cell response with tumour-infiltrating lymphocytes in these tumours induced by higher neoantigen exposure, an activated immune response, as well as a target for immune checkpoint inhibition, occurs. As much as nearly 20% of all colorectal tumours are considered to possess high TMB [22,23]. This demonstrates that high neoantigen expression is seen in some microsatellite stable tumours, such as those with base excision repair mutations or polymerase proofreading mutations, including POLE and POLD1 mutations [22].

### 2.2. Role of Immunotherapy in Serrated Adenocarcinoma of the Colon

Approximately 15–30% of colorectal cancers derive from the serrated pathway in contrast with the conventional adenoma–carcinoma sequence [24]. BRAF and KRAS mutant tumours have been shown to have a role in serrated biology [25]. BRAF initiating mutations can drive an MSI-H phenotype and often co-exists with MUC5AC mutations. However, the vast majority of serrated adenocarcinomas arise from MSS lesions. This is comprehensively reviewed elsewhere [26].

### 2.3. Consensus Molecular Subtypes in CRC

With the increasing understanding of the heterogeneity of colorectal cancer, the CRC consensus molecular subtype (CMS) classification has been agreed upon through the study of patients at multiple centres and whole transcriptomic analysis of tumour tissue [27]. CMS1 (“MSI Immune”), which represents 14% of patients, has high MSI with significant immune infiltration. Pathways affected include MMR and the PD-1/PD-L1 axis, with BRAF mutations. The most common subtype seen in 37% is CMS2 (“Canonical”), which, by contrast, has low MSI, but high somatic copy number alteration (SCNA) with mutations of P53 with Wnt/Myc signalling pathways affected. CMS3 (“Metabolic”) is only seen in 13% with mixed MSI status, and hypermethylation and metabolic pathways are affected. KRAS and PIC3CA mutations are observed in this group. Finally, CMS4 (“Mesenchymal”) subtype occurs in 23% of tumours, with low MSI, high somatic copy number alterations (SCNA), large stromal infiltration and driven by upregulation of TGF beta pathways, resulting in epidermal to mesenchymal transformation and extracellular matrix (ECM) remodelling. The CMS system aims to identify subtypes of CRC to stratify approaches based on phenotypes. It is worth highlighting that CMS1 and CMS4 have strong immune-related signatures. Whilst CMS1 is associated with MSI, increased T cell infiltration, and thus more likely to respond to immune checkpoint inhibitors, CMS4 is particularly immunosuppressive modulated by TGF beta upregulation. Combination therapy, which aims to activate and restore T-cell depletion whilst unlocking resistance pathways, may offer benefits in the setting of CMS4, where patients have the poorest prognosis.

### 2.4. Transcriptomic Profiling of Metastatic Colorectal Cancer

Further characterisation of not just primary colorectal tumours but also the metastatic lesion has given further insight into the complex biology of this disease. Pitroda and colleagues investigated molecular subtypes of colorectal liver metastases by combining CMS subtyping with mRNA and miRNA expression [28]. They concluded that in the metastatic setting, molecular features of the primary tumour were less prognostic and that analysis of the metastatic deposit differed and could more powerfully predict clinical outcomes based on three groups: canonical, immune and stromal subtypes. Differential genomic signatures between the primary tumour and the metastatic deposit have been demonstrated by other groups [29].

We believe that future approaches will weaponise emerging technology to characterise and define the tumour in patients with metastatic colorectal cancer, and this will guide initial therapy. Determining how systemic treatment changes the transcriptomic phenotype will maximise efficacy by adapting the personalised strategy to affect the T-cell response, as well as macrophage, neutrophil and ECM as collectively important components of the tumour microenvironment.

## 3. Colorectal Cancer and the Tumour Microenvironment—An Immunological Basis for Targeted Therapy

Most solid tumours generate some form of local inflammatory response. Under histological assessment, the invasion of adenocarcinoma through the muscularis and into the submucosa is met with immune cells at the invasive edge. On this battleground, resident and recruited cells aim to control and eliminate cancer cells through direct cytotoxicity, production of cytokines and through phagocytosis. However, colorectal cancer counters such mechanisms by the subversion of immune cells through apoptosis, converting cells to tumour-promoting phenotypes and adopting changes in surrounding stroma, inducing angiogenesis and ultimately, through migration and invasion. In patients with CMS1, with dMMR-MSI-H tumours, there is a high concentration of immune cells at the invasive edge [15]. It is likely that the degree of mutations resulting in high numbers of neoantigens on the tumoural surface is detected by resident immune cells and generates a robust immune response with recruitment and activation. As a result, such patients have a better prognosis [20]. Through the upregulation of PD1/PD-L1 pathways, tumours expressing higher levels of PD-L1 seem to have a worse prognosis, presumably through generating the “brakes” to achieve negative co-stimulation in surrounding immune cells and achieving immune privilege. By comparison, higher expression of PD-1/PD-L1 in the stromal immune cells appears to reflect active inflammation and is associated with better outcomes.

### 3.1. T-Cells

T-cells are traditionally considered part of the adaptive immune system. CD8 cells differentiate into CTL (cytotoxic T lymphocytes) after engagement with antigen-presenting cells (APC) to destroy tumour cells through perforin and granzyme granules. CD4 cells, T-helper-1 cells, support the response through cytokine production, such as IL-2 and IFN gamma, to promote T-cell maturation and activation of macrophages. Galon and colleagues showed that increased CD3 and CD8 T-cells at the tumour core and the invasive front, measurably by the Immunoscore, were associated with better prognosis [30]. Indeed, the infiltration of T-cells into the tumour itself is termed “tumour-infiltrating” lymphocytes (TILs) [31]. This observation suggests an activated cellular response and is associated with survival. However, increased expression of CTLA-4 interacts with CD80 and CD86 on APCs, preventing a co-stimulatory signal. PD-1 expression engages with PD-L1 on tumour cells as well as APCs causing a co-inhibitory signal and decreasing APC activation and promoting T-cell anergy and apoptosis.

T-regulatory (Treg) cells infiltrate the tumour microenvironment, which suppresses T-cell activation and the antitumoural response. Tregs produce suppressive cytokines, such as TGF beta, and express checkpoint proteins, such as PD-L1, LAG3 and CTLA4. Whilst increased numbers of CD3 and CD8 T-cells to the invasive front are associated with better outcomes in CRC, increased numbers of Tregs are associated with worse outcomes [31]. Additionally, CD4 T cells can differentiate into Th2, Th9, Th17, Th22, as well as T regulatory cells. Th2 and Th17 promote chronic inflammation, which hampers Th1 functions and promotes angiogenesis and immunosuppression. Additionally, along with NK cells and Th17 cells, Th22 cells secrete IL-22, which has a similar structure to IL-10, which promotes tumour progression. Associated STAT3 activation favours immunosuppressive functions of other immune cells, including dendritic cells and macrophages [32].

### 3.2. Natural Killer Cells

Natural Killer (NK) cells are a critical part of the innate immune response, which function to rapidly cause programmed cell death in cancerous cells lacking MHC I. Increased numbers of tumour-infiltrating NK cells, as well as T cells, in the specimens of resected colorectal liver metastases after neoadjuvant chemotherapy are predictive of overall survival [33].

### 3.3. Dendritic Cells

Dendritic cells (DCs) are resident, specialised APCs which coordinate the innate and adaptive responses. They act to promote tumour rejection through priming effect T-cells and phagocytose tumour antigens. DCs have been a target for vaccines against tumour antigens [34].

### 3.4. Myeloid Cells

Following the extravasation of circulating monocytes, these cells become macrophages. They are part of the innate response and are the centrepiece to cancer-associated inflammation [35,36]. Activated macrophages which occur through stimulation by IFN gamma, lipopolysaccharide (LPS) and TNF alpha, are termed M1 macrophages and are considered to be antitumoural in their role [37]. By comparison, M2 macrophages occur through exposure to IL-4 or IL-13 and are thought to be more tumourigenic. In later stages, tumour-associated macrophages (TAMs) tend to polarise towards an M2 phenotype which secrete IL-10 and TGF beta, promoting immunosuppression, impairing T cells and influencing the tumour microenvironment (TME) through angiogenesis (such as VEGF) and the extracellular matrix (such as through MMP9) [38]. Sites of metastases secrete CCL2 which attracts circulating monocytes to the metastatic tumour deposit.

Myeloid-derived suppressor cells (MDSCs) represent an immature population of bone marrow-derived cells which are broadly granulocytic (G-MDSC) or monocytic (M-MDSC). Following activation, immunosuppressive functions are acquired through upregulation of Arg1 and inducible nitrox oxide synthase (iNOS, and through CXCL1 from tumour cells, MDSCs express idoleamine 2,3 dixoygenase (IDO) within the TME promoting T cell apoptosis, inhibiting T cell function and suppressing T cell infiltration [32]. As a result, MDSCs are a major player in preventing the effectiveness of immunotherapy.

### 3.5. Neutrophils

Neutrophils are classically the first immune cell recruited to injury or inflammation. Tumour-associated neutrophils (TANs) demonstrate a similar phenotype in the early stage of cancer. These N1 TANs help educate T cells to reject tumours, promote tumour cell apoptosis through TRAIL and secrete reactive oxygen species (ROS). However, N2 TANs which are associated with later-stage cancer, driven by TGF beta, can promote angiogenesis, release MMP8 to degrade the ECM and secrete leukotrienes which expand tumour cells and worsen survival [32].

### 3.6. Translating Immunopathology Understanding into Therapeutics

**Figure 1:**
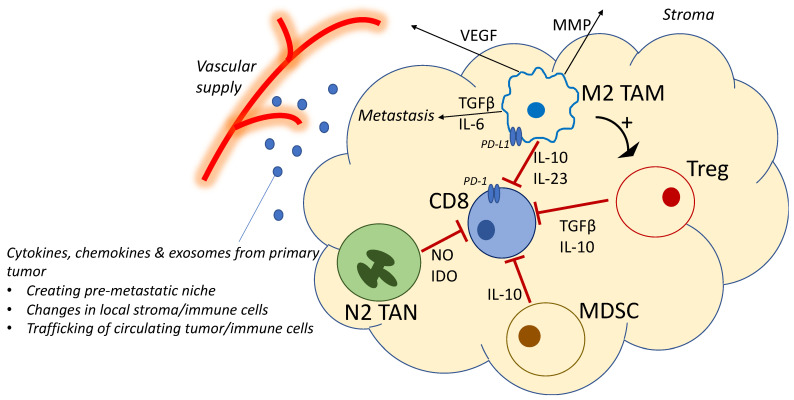
Cellular interactions in colorectal cancer metastasis. This proposed model illustrates the key changes in immune cells in an immunosuppressive tumour microenvironment, preventing surveillance, control and elimination of invading metastatic tumour cells. IDO, indoleamine 2,3-dioxygenase; IL, interleukin; MDSC, myeloid derived suppressor cells; MMP, matrix metalloproteinase; NO, nitric oxide; PD-1, programmed cell death receptor 1; PD-L1, programmed cell death receptor ligand 1; Treg, T regulatory cell; TAM, tumour associated macrophage; TAN, tumour-associated neutrophil; TGF, transforming growth factor; VEGF, vascular endothelial growth factor.

Thus, the effect of colorectal cancer-associated inflammation leads to dysregulation of multiple signalling pathways and stimulations, a chronic inflammatory process that influences the local immune cell populations and their function and phenotype. Failed antitumoural immunity and an immunosuppressive tumour microenvironment lead to the progress of the primary tumour and create a systemic pre-metastatic niche to promote the spread to local and distant sites. This is summarised in Figure 1. The changes in function described in the above section highlight an opportunity to interfere with this sequence of events, providing a natural role and rationale for immunotherapy.

Immune checkpoint inhibition has been the most successful form of immunotherapy for solid tumours. There has been strong evidence leading to a new era of therapy in malignant melanoma and lung cancer with the use of popular immune checkpoint inhibitors, which are being applied to colorectal cancer. The most promising results have been demonstrated in patients with deficient MMR status and MSI-H which is discussed in the next section. The most common immune checkpoint inhibitors in use and their molecular target are summarised in Table 1. A summary of prospective trials of immune checkpoint inhibitors is shown in Table 2 [39,40,41,42,43,44,45,46,47,48,49,50,51,52,53,54].

## 4. MMR Deficient—MSI-High Metastatic Colorectal Cancer

Immune checkpoint inhibition to target metastatic colorectal cancer was investigated by Le et al. in the Keynote-016 study. This phase I study of chemorefractory patients included those with both deficient and proficient MMR status who were treated with pembrolizumab [44]. In patients with deficient MMR status, this PD-1 inhibitor led to radiographic response rates in 33%, with up to 21% having a complete response rate. A follow-up report demonstrated a response rate of 40% in MMR deficient patients, at a 53% rate of progression-free survival (PFS) [41].

Further PD-1 inhibition using nivolumab was utilised in the Checkmate-142 study. Anti-PD-1 monotherapy was used in 74 patients with deficient MMR status [43]. An objective response was found in 31% of patients, with 69% of patients exhibiting disease control by a median follow-up of 12 months. Interestingly, immunohistochemistry of tumoural PD-L1 expression, KRAS and BRAF status were not predictive of response to immunotherapy. The most common side effect was fatigue and diarrhoea (in 22% of patients each), and less frequently hypothyroidism, hepatitis, arthralgia and pancreatitis.

A further cohort of the Checkmate-142 study investigated 119 patients undergoing combination therapy with anti-PD-1 and anti-CTLA-4 immunotherapy (nivolumab and ipilimumab, respectively). In this group of MMR deficient patients, response rates of 55% with disease control rates (DCR) of 80% were observed. Progression-free survival of 71% at 1 year was achieved, with overall survival (OS) at 85% at 1 year. The combination was more effective compared with nivolumab monotherapy (overall response rate of 55% compared with 31). However, 32% of patients experienced grade 3 or grade 4 side effects [42].

Further evidence for checkpoint inhibition in MMR deficient, microsatellite instability (MSI)-high patients was reported in the Keynote-164 study in 2020 [57]. One hundred twenty-four patients who had at least one previous line of therapy were recruited to this phase II study, 33% of which demonstrated a positive response to pembrolizumab. The first cohort of patients had >2 prior therapies and experienced a mean PFS of 2.3 months and median OS of 31.4 months. The second cohort, who only had >1 previous therapy, had a median PFS of 4.1 months and median OS, which was not reached by the time of analysis. Grade 3 or 4 side effects were seen in 16% and 13% of cohort A and B patients, respectively.

The Keynote-177 study was recently published in NEJM [21]. This phase III study included 307 MMR deficient–MSI-high patients who were treatment naïve. Pembrolizumab was compared with traditional chemotherapy (5FU with or without bevacizumab or cetuximab). Pembrolizumab was superior to chemotherapy with PFS of 16.6 months compared with 8.2 months, and the median OS has not yet been reached in the pembrolizumab group but was 36.7 months in the chemotherapy group based on the recent update [58]. An overall response rate was 43.8% in the pembrolizumab group, compared with 33.1% in the chemotherapy group. Importantly, only 22% of patients experienced grade 3 or 4 side effects in the pembrolizumab, compared with 66% in the chemotherapy group. Improved quality of life was also reported in the pembrolizumab monotherapy group in a follow-up report [59].

A Canadian study recently reported in JAMA Oncology investigated combination immunotherapy with tremelimumab and durvalumab compared with best supportive care in patients with metastatic refractory CRC [40]. In this phase II study (which included unselected patients based on MSI status), 180 patients were enrolled and found a median OS of 6.6 months in the immunotherapy group compared to 4.1 months in the supportive care group. A meagre improvement in PFS was seen with 1.8 months with immunotherapy compared with 1.9 months in the supportive care group. Up to 64% of patients experienced at least one grade 3 side effect related to immunotherapy. The authors concluded that OS was improved even in microsatellite stable metastatic CRC. Plasma measurements of tumour mutational burden (TMB) highlighted that increased TMB levels were associated with improved efficacy.

Thus, there is a rationale for upfront PD1 directed immunotherapy in MMR deficient patients. However, late-stage patients who are refractory to other chemotherapies fail to benefit.

## 5. MSS Metastatic Colorectal Cancer

The efficacy of immune checkpoint inhibitors has been far less successful in microsatellite stable (MSS) colorectal cancers. The Keynote-016 study discussed in the earlier section had included 18 patients with chemorefractory metastatic colorectal cancer and proficient MMR status were treated with pembrolizumab and found no responses in these patients [44].

Shahda and colleagues presented preliminary results of a phase II study combining pembrolizumab alongside chemotherapy (mFOLFOX6) irrespective of MMR status [60]. They found that of 30 patients enrolled, a complete response was seen in 1 patient, a partial response in 15 patients and 14 patients demonstrated stable disease. This is an ongoing study that was promising for MMR proficient patients.

Due to the poor results seen in immune checkpoint inhibition, this has led to a number of studies using various combination strategies aiming to improve the efficacy of immunotherapy in MMR proficient metastatic colorectal cancer [61]. Combination strategies will be discussed in the following section.

## 6. Combination Strategies in Metastatic Colorectal Cancer—Overcoming Resistance

In patients with microsatellite instable-MMR deficient metastatic colorectal cancer, the high tumour mutational burden with high neoantigen rates creates increased MHC I expression and increased numbers of infiltrating CD8 T-cells. The concept of combining immunotherapy with an additional modality attempts to synergistically stimulate the tumour microenvironment to increase interferon-gamma and other cytokines and promote immune cell recruitment.

### 6.1. Immunotherapy and MEK Inhibitors

In order to assess the role of atezolizumab alongside traditional chemotherapy, the MODUL trial used a biomarker-driven approach [62]. Following standard induction across groups with FOLFOX and bevacizumab, depending on the status of BRAF, MSI and HER2, different regimes were trialled compared with 5FU or capecitabine with bevacizumab. Atezolizumab was used alongside patents a fluoropyrimidine/bevacizumab if MSI high was identified, or alongside cobimetanib in one of the umbrella cohorts. In BRAF wild-type patients, the use of bevacizumab is hoped to increase T cell trafficking whilst reducing Treg/MDSC infiltration and thus improve immune checkpoint efficacy. Similarly, the combination with a MEK inhibitor aims to sensitise tumours to atezolizumab. Final results are eagerly awaited.

A MEK inhibitor (cobimetanib) was used in combination with atezolizumab in the IMblaze 370 trial [54]. This included 363 patients with unresectable or metastatic colorectal cancer and compared the combination of MEK inhibitor and PD-L1 inhibitor against PD-L1 inhibitor alone or regorafenib (EGFR/VEGF inhibitor). Patients had microsatellite stable disease in 93%, 92% and 89% in each group, respectively. The authors reported that median overall survival was 8.87 months using the combination, compared with 2.1 months with atezolizumab and 8.51 months with regorafenib. The lack of benefit using immunotherapy, with or without the combination of a MEK inhibitor, reinforced the poor responses of immunotherapy in patients with low levels of inflammation.

The combination of cobimetanib and atezolizumab was reported by Bendell and colleagues in a phase 1b trial in 2016 [63]. This suggested responses in some MSS colorectal cancer irrespective of PD-L1 expression, with responders showing upregulated PD-L1 expression and T-cell infiltration. Subsequently, Hellmann and colleagues reported a response rate in seven patients (8%) of the cohort, of whom six patients had microsatellite stable status [64].

### 6.2. Immunotherapy and CEA Inhibition

Targeting carcinogenic embryonic antigen on tumour cells and CD3 T cells is under investigation which aims to promote T cell infiltration and upregulate PD-L1 [65]. A phase I dose-escalation study compares this agent as monotherapy against a combination of CEA (carcinoembryonic antigen) antibody and atezolizumab in both MSS and MSI patients. The authors report that at high doses, many patients demonstrated tumour inflammation and a partial response seen in 5% of patients receiving monotherapy and 20% of those receiving the combination with immune checkpoint inhibition. Interestingly, all patients had tumours which were microsatellite stable.

### 6.3. Immunotherapy and IDO Inhibitors

The immunosuppressive enzyme idoleamine 2,3-dioxygenase (IDO) has been a target of inhibition in combination with nivolumab in the ECHO-204 study [66]. This included 241 patients with various advanced solid cancers and was performed as a phase 1/2 dose-escalation study. In the 25 colorectal cancer patients, overall response rates were 4% and disease control rates of 24%.

### 6.4. Immunotherapy and Radiotherapy

As immune checkpoint inhibitors have low efficacy alone in patients with proficient MMR metastatic colorectal cancer, investigators are studying the potential combination of radiotherapy (RT) and immunotherapy. Segal and colleagues studied 26 patients with unresectable or recurrent metastatic colorectal cancer who have already failed at least two standard therapies [67]. They received palliative RT or ablation to the lesion (this varied between colorectum, liver, lung or bone) along with pembrolizumab. Interim overall response rates were 9% in patients receiving radiotherapy, with no responses in the ablation group. The hypothesis by combining radiotherapy with immunotherapy is that irradiation will induce a local inflammatory response and thus upregulation of PD1/PD-L1 pathways, which will improve the efficacy of immune checkpoint inhibition. This is under investigation in the PRIME-RT trial in patients with locally advanced rectal cancer, which includes patients with both proficient and deficient MMR status undergoing durvalumab alongside extended neoadjuvant regimes (NCT04621370).

The Checkmate-142 study, which was discussed earlier in this review, also included additional treatment arms utilising combinations of immunotherapy. This included nivolumab and anti-LAG3 antibodies (another co-inhibitory protein), and combination with nivolumab, ipilimumab and cobimetanib (MEK inhibitor) and nivolumab with daratumumab (anti-CD38 antibody)—a protein previously associated with tumour cells of myeloma.

## 7. Preclinical Studies Investigating Promising Immunotherapy Strategies in Metastatic Colorectal Cancer

New targets for therapy continue to be identified via ongoing translational research to identify underpinning molecular mechanisms of immunopathology. Macrophages within the tumour microenvironment have been identified as key players in tumour progression, where an M2 phenotype creates resistance to anti-PD-1/PD-L1 agents [38]. In preclinical models, CSF1 receptor blockade has been effective in polarisation to M1 macrophages and restoring effective immune checkpoint immunotherapy. This is now under investigation in both pancreatic and colorectal cancer using a combination of durvalumab and pexidartinib (anti-CSF1R) (NCT02777710).

Other emerging therapies include antibodies targeting OX40 and CD73 immunotherapy. OX40 (CD134) is a member of the TNF receptor superfamily with a role in co-stimulatory immune responses, which are expressed on T-cells. This interacts with OX40 ligand on antigen-presenting cells resulting in activation of NFKB and NFAT. Given the high levels expressed on Tregs and increases in the generation of those and similar immunosuppressive T cell subsets, OX40 inhibition aims to counteract tumour-infiltrating Tregs and thus aims to improve immune checkpoint efficacy [68]. CD73 is a novel immune checkpoint protein associated with tumour progression and suppressing antitumoural immune responses. Combination with CD73 monoclonal antibodies and immune checkpoint inhibitors is under clinical investigation for solid organ tumours [69]. There are many more examples of novel targets relating to immunotherapy that are beyond the scope of this review.

## 8. Genomic Biomarkers of Response to Immunotherapy

A difficulty remains in defining which patients will respond best to immunotherapy. Consistently across different tumour types, tumour mutational burden is a strong predictor, particularly in melanoma and non-small cell lung cancer [70]. The more somatic mutations a tumour has, the more likely it is to present neoantigens. However, it is dependent on the mutation whether a peptide is loaded onto the MHC and presented to T cells, with only a minority being presented in this fashion [71]. Amongst colorectal cancer, mutational burden has proven to be an excellent predictor of response to ICB in MSI-H patients [72], whilst it is acknowledged that certain MSS patients have a high mutational burden, but these numbers, when studied, are in the order of 164/5704 [73]. However, Alexandrov et al. took this further by examining the influence of “characteristic mutational processes” on cancer. Through detailed examination of somatic mutations obtained from registered genome sequences, the authors demonstrated the ability to derive multiple new single base substitution signatures, small insertions and deletions and double base signatures [74]. These data will, in the future, yield information on how these processes lead to carcinogenesis and are processed by the immune system. Indeed, for example, the identification of the APOBEC mutational signature has shown some promise in NSCLC in combination with mutational burden in identifying patients that respond better to ICB [75]. Patients demonstrated higher levels of effector immune cell infiltration to their tumours and reduction in regulatory T cells. Patterns of mutations in MSS CRC may include novel DNA damage mutations, including ATM, which can sensitise patients to durable responses to ICB [76]. Increasingly advanced techniques of pattern recognition are being explored in this field with advances in digital pathology. MSI status, though now universally tested, can be predicted using deep learning algorithms and artificial intelligence from histology [77]. Additionally, common actionable mutations can be detected in a similar fashion, and this technology holds hope for the development of personalised treatment algorithms [78].

## 9. Strategies to Improve Outcomes

Many basic science and clinical researchers are exploring different avenues in order to make stepwise gains in the advancement of care for patients with metastatic colorectal cancer. New technologies allow for better characterisation of the genomic and molecular mechanisms underpinning colorectal cancer and the pathological responses of the local and systemic immune components. More realistic preclinical models using autochthonous models, organoids and patient-derived xenografts in animals permit the study of novel agents which target not just inflammatory pathways but also have potential to reprogramme pro-tumourigenic pathways regulated by cancer-associated lymphocytes, macrophages, neutrophils and cancer-associated fibroblasts.

Tauriello and colleagues adopted a model of murine metastatic colorectal cancer with APC, p53, KRAS and TGF-β mutations [79]. This produced a microsatellite-stable phenotype that had limited responses to PD-1/PD-L1 inhibition but revealed a TGFβ-driven suppression of T-cells and specifically TH1 cells. Jackstadt et al. further established using an autochthonous model of serrated adenocarcinoma that TGFβ signalling drove neutrophilic infiltration to spontaneous liver metastases, a phenotype that was inhibited through neutrophil and TGFβ inhibition [80]. Given the reproducible nature of patient-derived organoids and patient-derived xenografts and their representative therapeutic responses in patients, they have the power to become a tool to investigate novel immunotherapeutics for patients with metastatic colorectal cancer. Engraftment of colorectal cancer to immunocompetent mice using genetically engineered mice organoids, wild-type organoids or patient-derived human CRC organoids can be used to study in vivo immunological interactions in the setting of metastatic colorectal cancer [81]. In a world with increasing challenges in clinical academia due to the coronavirus pandemic, supporting the discovery and translation of new approaches in armouring and modifying the immune response is critical. One example of this is the Accelerator Award from CRUK, which aims to support infrastructures, such as sharing datasets, models and tissue biobanks and support facets of oncology research, such as preclinical studies, biomarkers, imaging, radiotherapy and engineering approaches [82]. Immunotherapies other than immune checkpoint inhibitors, which have been studied, were identified in our literature search and are summarised in Supplementary Table S1.

The influence of gut microbiota on the tumour microenvironment and, therefore, responses to immunotherapy are wide-ranging, varied, and yet to be fully explored. The microbiome in the gut is predominated by anaerobes, different species of whom have been shown to have influences on different immune cells within tumours, in addition to interacting directly with immune checkpoints. These interactions are thoroughly described in previous review material [83]. Personalised responses to ICB (immune checkpoint blockade) are a result of this heterogeneity influenced by gut flora. Whilst enterococcus faecium and klebsiella pneumonia are common gut pathogens associated with positive responses to ICB, patients with high numbers of lactobacillus have poorer responses. It is increasingly likely as trials develop, particularly in MSS colorectal cancer, that microbial species in faeces may be first studied to help predict responses and altered to enhance efficacy.

**Figure 2:**
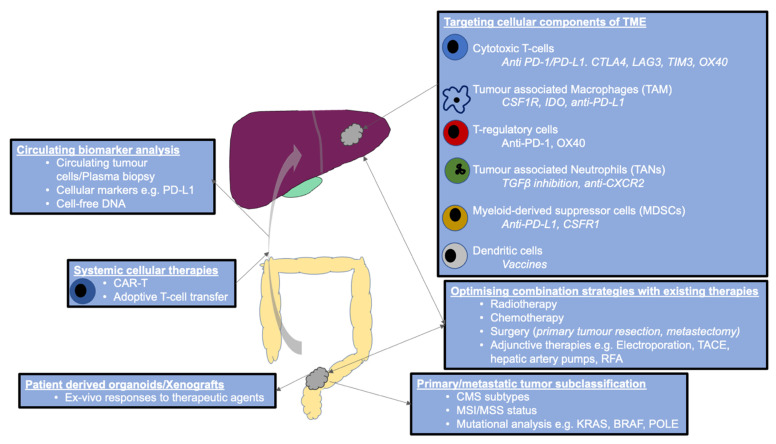
Proposed model for immunotherapy in metastatic colorectal cancer. TME, tumour microenvironment; PD-1, programmed cell death receptor 1; PD-L1, programmed cell death receptor ligand 1; CTLA4, T-lymphocyte associated protein 4; LAG3, lymphocyte activating gene 3; TIM3, T cell immunoglobulin mucin receptor 3; CSF1R, colony-stimulating factor 1 receptor; IDO, indoleamine 2,3-dioxygenase; TGF, transforming growth factor; CAR-T, chimeric antigen receptor T-cell; TACE, transhepatic arterial chemoembolisation; RFA, radiofrequency ablation; KRAS, Kirsten rat associated sarcoma; BRAF, B-type RAF; POLE, DNA polymerase epsilon.

Patients with metastatic colorectal disease range from those with low burden oligometastatic hepatic disease to overwhelming, aggressive widespread metastases. Given the range of colorectal cancer subtypes based on the location of primary and CMS phenotypes, then it is likely that it will take different strategies to improve survival for stratified patient groups (Figure 2). For example, using immunotherapy as an adjunct in the pre- or post-operative phases, will benefit patients with potentially curative metastatic colorectal cancer, which is very different from identifying molecular subtypes amenable to new immunotherapies to achieve better palliation or partial responses in certain subsets of widespread metastatic disease in patients that present with palliative disease.

## 10. Conclusions

Metastatic colorectal cancer continues to have a dismal prognosis and is a major cause of death in the Western world. Established regimes for chemotherapy have relatively poor rates of response yet cause significant side effects which impair quality of life in end-stage disease. Immunotherapy represents a new dawn of therapeutics, which could not only improve the balance of benefit and side effect profile but also may offer better responses in certain patient groups. We have discussed the goals of the clinical and scientific community in discovering how to translate the benefits and efficacy of immune checkpoint inhibitors and other novel agents to the right groups of patients.

## Figures and Tables

**Table 1:** Types of immune checkpoint inhibitors.

Classification	Name	Trade Name
Anti PD-1	Pembrolizumab	Keytruda
	Nivolumab	Opdivo
	Atezolizumab	Tecentriq
Anti PD-L1	Durvalumab	Imfinzi
	Avelumab	Bavencio
Anti CTLA4	Ipilimumab	Yervoy
	Tremelimumab	N/A

**Table 2:** Prospective studies of immune checkpoint inhibitors in metastatic colorectal cancer.

First Author	Journal	Year	Type	Target	Patient Selection	Generic	Type	Key Findings
Chung [47]	J Clin Oncol.	2010	Immune checkpoint inhibitor	CTLA4	All patients	Tremelimumab	Phase II	Response rate 27% for nivolumab only, and 15% in nivolumab plus ipilimumab.
Topalian [48]	NEJM	2012	Immune checkpoint inhibitor	PD-1	Includes NSCLC, MM, RCC, prostate ca and CRC.	BMS-936558	Phase I	No clear benefit but one patient with partial response.
Brahmer [49]	NEJM	2012	Immune checkpoint inhibitor	PD-L1	Includes CRC, RCC, ovarian ca, pancreatic ca, gastric ca, breast ca		Phase I	No objective responses in patients with CRC.
Le [44]	NEJM	2015	Immune checkpoint inhibitor	PD-1	Both dMMR and pMMR	Pembrolizumab	Phase II	Response rates at 31% by 12 months, with 69% disease control rate of 3 months or longer.
Bendell [55]	J Clin Oncol.	2015	Immune checkpoint inhibitor/bevacizumab or FOLFOX	PD-L1	All patients	Atezolizumab	Phase 1b	No objective responses in patients with CRC.
Overman [46]	J Clin Oncol.	2016	Immune checkpoint inhibitor	PD-1/CTLA4	All patients	Nivolumab and ipilimumab	Phase II	Adverse events occurred early, were manageable, and did not affect outcome
Bendell [56]	J Clin Oncol.	2016	Immune checkpoint inhibitor/MEK inhibitor	PD-L1/MEK	All patients	Atezolizumab	Phase 1b	Response rates were 8% for anti-PD-L1/bev, compared with 36% in patients with anti-PD-L1/bev/FOLFOX6
Le [41]	Science	2017	Immune checkpoint inhibitor	PD-1	High MSI/dMMR	Pembrolizumab	Phase II	Prolonged OS in advanced refractory CRC.
Overman [43]	Lancet Oncol.	2017	Immune checkpoint inhibitor	PD-1	All patients	Nivolumab	Phase II	Response rates at 55%, with disease control rates for more than 3 months in 80%.
Overman [42]	J Clin Oncol.	2018	Immune checkpoint inhibitor	PD-1/ CTLA4	High MSI/dMMR	Nivolumab and ipilimumab	Phase II	Responses in 53% patients, with complete responses in 21%.
Morse [45]	Oncologist	2019	Immune checkpoint inhibitor	PD-1/ CTLA4	High MSI/dMMR	Nivolumab and ipilimumab	Phase II	Response rate 40% in dMMR and 0% in pMMR
Mettu [51]	Annals of Oncol.	2019	Immune checkpoint inhibitor/ capectabine/VEGF	PD-L1/ CTLA4	All patients	Atezolizumab	Phase II	No patients had a tumour response.
Eng C [54]	Lancet Oncol.	2019	Immune checkpoint inhibitor/ MEK/VEGFR2	PD-L1/ MEK/VEGFR2	All patients	Atezolizumab	Phase III	No response, disease control rate in 78%, progression in 22%.
Antoniotti [39]	BMC Cancer	2020	Immune checkpoint inhibitor/ FOLFOXIRI/ bevacizumab	PD-L1	All patients	Atezolizumab	Phase II	Combination strategy appears safe. Ongoing enrolment.
Chen [40]	JAMA Oncol.	2020	Immune checkpoint inhibitor	PD-L1/ CTLA4	All patients	Durvalumab and Tremelimumab	Phase II	No major safety concerns. Ongoing enrolment.
Patel [50]	Cancer Medicine	2020	Immune checkpoint inhibitor/ trifluridine/tipiracil	PD-1	MSS	Nivolumab	Phase II	Overall response rate 17%, not associated PD-L1 expression.
Li [53]	Frontiers in Oncol.	2020	Immune checkpoint inhibitor/RTK	PD-1/ VEGFR2	MSS/pMMR	Mixture	Retrospective	Response rate 8%.
Andre [21]	NEJM	2020	Immune checkpoint inhibitor	PD-1	MSI-H/dMMR	Pembrolizumab	Phase III	Response rate 44% vs. 33% (chemo), improved PFS.
Segal [52]	Clinical Cancer Res.	2021	Immune checkpoint inhibitor/RT	PD-L1/ CTLA4	pMMR	Durvalumab and Tremelimumab	Phase II	Anti-PD-L1 added to capecitabine and bevacizumab improves response rates from 4 to 8%.

## Data Availability

There was no original data reported in this study.

## Supplementary Materials

The following are available online at https://www.mdpi.com/article/10.3390/cancers13143566/s1, Table S1: Other immunotherapy strategies in metastatic colorectal cancer.
