# Supplementary material for: Targeting Metastatic Colorectal Cancer with Immune Oncological Therapies
Source: Cancers (Basel). 2021 Jul 16;13(14):3566. doi: 10.3390/cancers13143566 (PMC8307556; doi:10.3390/cancers13143566)
Supplement: Supplementary file 1 [file cancers-13-03566-s001.zip › cancers-1259741-supplementary.pdf]

cancers

MDPI

Review

Targeting Metastatic Colorectal Cancer with Immune Oncological Therapies

Norman J. Galbraith, Colin Wood and Colin W. Steele

Table S1. Other immunotherapy strategies in metastatic colorectal cancer.

**Vaccines**				
Speetjens FM **[1]**	Induction of p53-specific immunity by a p53 synthetic long peptide vaccine in patients treated for metastatic colorectal cancer	Clin Cancer Res	2009	Vaccine
Neidhart J **[2]**	Immunization of colorectal cancer patients with recombinant baculovirus-derived KSA (Ep-CAM) formulated with monophosphoryl lipid A in liposomal emulsion, with and without granulocyte-macrophage colony-stimulating factor	Vaccine	2004	Vaccine
Samonigg H **[3]**	A double-blind randomized-phase II trial comparing immunization with antiidiotype goat antibody vaccine SCV 106 versus unspecific goat antibodies in patients with metastatic colorectal cancer	J Immunother	1999	Vaccine
Schimanski CC **[4]**	LICC: L-BLP25 in patients with colorectal carcinoma after curative resection of hepatic metastases: a randomized, placebo-controlled, multicenter, multinational, double-blinded phase II trial	BMC Cancer	2012	Vaccine
Schulze T **[5]**	Efficiency of adjuvant active specific immunization with Newcastle disease virus modified tumor cells in colorectal cancer patients following resection of liver metastases: results of a prospective randomized trial	Cancer Immunol Immunother	2009	Vaccine
Hoover HC Jr **[6]**	Delayed cutaneous hypersensitivity to autologous tumor cells in colorectal cancer patients immunized with an autologous tumor cell: Bacillus Calmette-Guérin vaccine	Cancer Res	1984	Vaccine
	**Other immunotherapies**			
Weihrauch MR **[7]**	Phase I/II combined chemoimmunotherapy with carcinoembryonic antigen-derived HLA-A2-restricted CAP-1 peptide and irinotecan, 5-fluorouracil, and leucovorin in patients with primary metastatic colorectal cancer	Clin Cancer Res	2005	CEA peptide
Van Cutsem E **[8]**	An open-label, single-arm study assessing safety and efficacy of panitumumab in patients with metastatic colorectal cancer refractory to standard chemotherapy	Ann Oncol	2008	EGFR Mab
Taniguchi H **[9]**	Phase 1 study of OCV-C02, a peptide vaccine consisting of two peptide epitopes for refractory metastatic colorectal cancer	Cancer Sci	2017	Vaccine
Rubin J **[10]**	Phase I study of immunotherapy of hepatic metastases of colorectal carcinoma by direct gene transfer	Hum Gene Ther	1994	
Caballero-Baños M **[11]**	Phase II randomised trial of autologous tumor lysate dendritic cell plus best supportive care compared with best supportive care in pre-treated advanced colorectal cancer patients	Eur J Cancer	2016	
Peethambaram PP **[12]**	A phase I trial of immunotherapy with lapuleucel-T (APC8024) in patients with refractory metastatic tumors that express HER-2/neu	Clin Cancer Res	2009	
MacLean GD **[13]**	Antibodies against mucin-associated sialyl-Tn epitopes correlate with survival of metastatic adenocarcinoma patients undergoing active specific immunotherapy with synthetic STn vaccine	J Immunother.	1996	

Morse MA **[14]**	A randomized phase II study of immunization with dendritic cells modified with poxvectors encoding CEA and MUC1 compared with the same poxvectors plus GM-CSF for resected metastatic colorectal cancer	Ann Surg	2013	Vaccine
Moulton HM **[15]**	Active specific immunotherapy with a beta-human chorionic gonadotropin peptide vaccine in patients with metastatic colorectal cancer: antibody response is associated with improved survival	Clin Cancer Res	2002	Vaccine
Scurr M **[16]**	Effect of Modified Vaccinia Ankara-5T4 and Low-Dose Cyclophosphamide on Antitumor Immunity in Metastatic Colorectal Cancer: A Randomized Clinical Trial	JAMA Oncol	2017	Vaccine
Zhao H **[17]**	Autologous Cytokine-Induced Killer Cells Improves Overall Survival of Metastatic Colorectal Cancer Patients: Results From a Phase II Clinical Trial	Clin Colorectal Cancer	2016	Cell therapy
	**Cytokine immunotherapy**			
Nichols PH **[18]**	Perioperative immunotherapy with recombinant interleukin 2 in patients undergoing surgery for colorectal cancer	Cancer Res	1992	IL2
Elkahwaji J **[19]**	Decrease in hepatic cytochrome P450 after interleukin-2 immunotherapy	Biochem Pharmacol	1999	IL2
Palmeri S **[20]**	5-Fluorouracil plus interferon alpha-2a compared to 5-fluorouracil alone in the treatment of advanced colon carcinoma: a multicentric randomized study	J Cancer Res Clin Oncol	1998	IFN alpha
Fu QG **[21]**	Efficacy of intraperitoneal thermochemotherapy and immunotherapy in intraperitoneal recurrence after gastrointestinal cancer resection	World J Gastroenterol	2002	IL2

References

1. Speetjens, F.M.; Kuppen, P.J.; Welters, M.J.; Essahsah, F.; Brink, A.M.V.v.d.; Lantrua, M.G.; Valentijn, A.R.; Oostendorp, J.; Fathers, L.M.; Nijman, H.W.; et al. Induction of p53-specific immunity by a p53 synthetic long peptide vaccine in patients treated for metastatic colorectal cancer. *Clin. Cancer Res.* **2009**, *15*, 1086–1095, doi:10.1158/1078-0432.Ccr-08-2227.
2. Neidhart, J.; Allen, K.O.; Barlow, D.L.; Carpenter, M.; Shaw, D.R.; Triozzi, P.L.; Conry, R.M. Immunization of colorectal cancer patients with recombinant baculovirus-derived KSA (Ep-CAM) formulated with monophosphoryl lipid A in liposomal emulsion, with and without granulocyte-macrophage colony-stimulating factor. *Vaccine* **2004**, *22*, 773–780, doi:10.1016/j.vaccine.2003.08.021.
3. Samonigg, H.; Wilders-Truschnig, M.; Kuss, I.; Plot, R.; Stöger, H.; Schmid, M.; Bauernhofer, T.; Tiran, A.; Pieber, T.; Havelec, L.; et al. A double-blind randomized-phase II trial comparing immunization with antiidiotype goat antibody vaccine SCV 106 versus unspecific goat antibodies in patients with metastatic colorectal cancer. *J. Immunother.* **1999**, *22*, 481–488.
4. Schimanski, C.C.; Möhler, M.; Schön, M.; van Cutsem, E.; Greil, R.; Bechstein, W.O.; Hegewisch-Becker, S.; von Wichert, G.; Vöhringer, M.; Heike, M.; et al. LICC: L-BLP25 in patients with colorectal carcinoma after curative resection of hepatic metastases: A randomized, placebo-controlled, multicenter, multinational, double-blinded phase II trial. *BMC Cancer* **2012**, *12*, 144, doi:10.1186/1471-2407-12-144.
5. Schulze, T.; Kemmner, W.; Weitz, J.; Wernecke, K.D.; Schirrmacher, V.; Schlag, P.M. Efficiency of adjuvant active specific immunization with Newcastle disease virus modified tumor cells in colorectal cancer patients following resection of liver metastases: Results of a prospective randomized trial. *Cancer Immunol. Immunother.* **2009**, *58*, 61–69, doi:10.1007/s00262-008-0526-1.
6. Hoover, H.C., Jr.; Surdyke, M.; Dangel, R.B.; Peters, L.C.; Hanna, M.G., Jr. Delayed cutaneous hypersensitivity to autologous tumor cells in colorectal cancer patients immunized with an autologous tumor cell: Bacillus Calmette-Guérin vaccine. *Cancer Res.* **1984**, *44*, 1671–1676.
7. Weihrauch, M.R.; Ansén, S.; Jurkiewicz, E.; Geisen, C.; Xia, Z.; Anderson, K.S.; Gracien, E.; Schmidt, M.; Wittig, B.; Diehl, V.; et al. Phase I/II combined chemoimmunotherapy with carcinoembryonic antigen-derived HLA-A2-restricted CAP-1 peptide and irinotecan, 5-fluorouracil, and leucovorin in patients with primary metastatic colorectal cancer. *Clin. Cancer Res.* **2005**, *11*, 5993–6001, doi:10.1158/1078-0432.Ccr-05-0018.
8. Van Cutsem, E.; Siena, S.; Humblet, Y.; Canon, J.L.; Maurel, J.; Bajetta, E.; Neyns, B.; Kotasek, D.; Santoro, A.; Scheithauer, W.; et al. An open-label, single-arm study assessing safety and efficacy of panitumumab in patients with metastatic colorectal cancer refractory to standard chemotherapy. *Ann. Oncol.* **2008**, *19*, 92–98, doi:10.1093/annonc/mdm399.
9. Taniguchi, H.; Iwasa, S.; Yamazaki, K.; Yoshino, T.; Kiryu, C.; Naka, Y.; Liew, E.L.; Sakata, Y. Phase 1 study of OCV-C02, a peptide vaccine consisting of two peptide epitopes for refractory metastatic colorectal cancer. *Cancer Sci.* **2017**, *108*, 1013–1021, doi:10.1111/cas.13227.

10. Rubin, J.; Galanis, E.; Pitot, H.C.; Richardson, R.L.; Burch, P.A.; Charboneau, J.W.; Reading, C.C.; Lewis, B.D.; Stahl, S.; Akporiaye, E.T.; et al. Phase I study of immunotherapy of hepatic metastases of colorectal carcinoma by direct gene transfer of an allogeneic histocompatibility antigen, HLA-B7. *Gene Ther.* **1997**, *4*, 419–425, doi:10.1038/sj.gt.3300396.
11. Caballero-Baños, M.; Benitez-Ribas, D.; Tabera, J.; Varea, S.; Vilana, R.; Bianchi, L.; Ayuso, J.R.; Pagés, M.; Carrera, G.; Cuatrecasas, M.; et al. Phase II randomised trial of autologous tumour lysate dendritic cell plus best supportive care compared with best supportive care in pre-treated advanced colorectal cancer patients. *Eur. J. Cancer* **2016**, *64*, 167–174, doi:10.1016/j.ejca.2016.06.008.
12. Peethambaram, P.P.; Melisko, M.E.; Rinn, K.J.; Alberts, S.R.; Provost, N.M.; Jones, L.A.; Sims, R.B.; Lin, L.R.; Frohlich, M.W.; Park, J.W. A phase I trial of immunotherapy with lapuleucel-T (APC8024) in patients with refractory metastatic tumors that express HER-2/neu. *Clin. Cancer Res.* **2009**, *15*, 5937–5944, doi:10.1158/1078-0432.Ccr-08-3282.
13. MacLean, G.D.; Reddish, M.A.; Koganty, R.R.; Longenecker, B.M. Antibodies against mucin-associated sialyl-Tn epitopes correlate with survival of metastatic adenocarcinoma patients undergoing active specific immunotherapy with synthetic STn vaccine. *J. Immunother. Emphasis Tumor Immunol.* **1996**, *19*, 59–68, doi:10.1097/00002371-199601000-00007.
14. Morse, M.A.; Niedzwiecki, D.; Marshall, J.L.; Garrett, C.; Chang, D.Z.; Aklilu, M.; Crocenzi, T.S.; Cole, D.J.; Dessureault, S.; Hobeika, A.C.; et al. A randomized phase II study of immunization with dendritic cells modified with poxvectors encoding CEA and MUC1 compared with the same poxvectors plus GM-CSF for resected metastatic colorectal cancer. *Ann. Surg.* **2013**, *258*, 879–886, doi:10.1097/SLA.0b013e318292919e.
15. Moulton, H.M.; Yoshihara, P.H.; Mason, D.H.; Iversen, P.L.; Triozzi, P.L. Active specific immunotherapy with a beta-human chorionic gonadotropin peptide vaccine in patients with metastatic colorectal cancer: Antibody response is associated with improved survival. *Clin. Cancer Res.* **2002**, *8*, 2044–2051.
16. Scurr, M.; Pembroke, T.; Bloom, A.; Roberts, D.; Thomson, A.; Smart, K.; Bridgeman, H.; Adams, R.; Brewster, A.; Jones, R.; et al. Effect of Modified Vaccinia Ankara-5T4 and Low-Dose Cyclophosphamide on Antitumor Immunity in Metastatic Colorectal Cancer: A Randomized Clinical Trial. *JAMA Oncol.* **2017**, *3*, e172579, doi:10.1001/jamaoncol.2017.2579.
17. Zhao, H.; Wang, Y.; Yu, J.; Wei, F.; Cao, S.; Zhang, X.; Dong, N.; Li, H.; Ren, X. Autologous Cytokine-Induced Killer Cells Improves Overall Survival of Metastatic Colorectal Cancer Patients: Results From a Phase II Clinical Trial. *Clin. Colorectal. Cancer* **2016**, *15*, 228–235, doi:10.1016/j.clcc.2016.02.005.
18. Nichols, P.H.; Ramsden, C.W.; Ward, U.; Sedman, P.C.; Primrose, J.N. Perioperative immunotherapy with recombinant interleukin 2 in patients undergoing surgery for colorectal cancer. *Cancer Res.* **1992**, *52*, 5765–5769.
19. Elkahwaji, J.; Robin, M.A.; Berson, A.; Tinel, M.; Lettéron, P.; Labbe, G.; Beaune, P.; Elias, D.; Rougier, P.; Escudier, B.; et al. Decrease in hepatic cytochrome P450 after interleukin-2 immunotherapy. *Biochem. Pharmacol.* **1999**, *57*, 951–954, doi:10.1016/s0006-2952(98)00372-4.
20. Palmeri, S.; Meli, M.; Danova, M.; Bernardo, G.; Leonardi, V.; Dastoli, G.; Rausa, L.; Russo, A.; Filippelli, G.; Palmieri, G.; et al. 5-Fluorouracil plus interferon alpha-2a compared to 5-fluorouracil alone in the treatment of advanced colon carcinoma: A multicentric randomized study. *J. Cancer Res. Clin. Oncol.* **1998**, *124*, 191–198, doi:10.1007/s004320050154.
21. Fu, Q.G.; Meng, F.D.; Shen, X.D.; Guo, R.X. Efficacy of intraperitoneal thermochemotherapy and immunotherapy in intraperitoneal recurrence after gastrointestinal cancer resection. *World J. Gastroenterol.* **2002**, *8*, 1019–1022, doi:10.3748/wjg.v8.i6.1019.
